# Richard Tregear, Co-founder of the Journal of Muscle Research and Cell Motility

**DOI:** 10.1007/s10974-021-09600-2

**Published:** 2021-03-11

**Authors:** Steven Baxter Marston

**Affiliations:** Imperial College London, London, UK

Richard Tregear died 1st May 2020, he was 88 years old.

He will be remembered as a significant contributor to our understanding of muscle contraction, particularly in the 1960s and 1970s but more especially as a cofounder of The Journal of Muscle Research and Cell Motility.

Richard studied physics at Cambridge and after spells as a schoolteacher and as a researcher at Porton Down, he was picked by John Pringle to join his newly-formed ARC Unit of Insect Physiology in the Zoology Department of Oxford University. This talented group contained a potent mixture of physicsists, biologists, physiologists and biochemists that included Eric Newsholme, Belinda Bullard, Roger Abbot and Peter Brunet. The fundamental research done by this group exploited the unique properties of the fibrillar flight muscle of the giant water bug Lethocerus.

Richard’s research contributions were seminal in many areas and include the first direct demonstration of stretch-activation of actomyosin ATPase (Rüegg and Tregear 1966) and the direct visualisation of rigor and relaxed crossbridges in muscle by EM and X-ray techniques (Miller and Tregear 1970; Reedy et al. 1965; Tregear and Miller 1969); this work was roughly contemporaneous with Hugh Huxley’s studies but gained much less recognition.

He was a modest and very thoughtful man who revelled in scientific arguments; he was closely involved in the challenges and controversies of this exciting era of muscle research. He achieved much by asking questions and motivating others to find answers rather than as an individual experimenter. Amongst the research he instigated were the demonstration that the Myosin ADP.Pi complex, predicted by in vitro kinetics, was present in muscle (Marston and Tregear 1972), that substrate binding and mechanical output were directly and reversibly coupled (Marston et al. 1979), that the proportion of myosin to actin varied between muscle types and was directly related to the way the filaments were arranged (Tregear and Squire 1973) (this was a pioneering quantitative gel electrophoresis study) and he made the first X-ray diffraction measurements of the temporal relationship of crossbridge movement to muscle force by taking advantage of the cyclical nature of stretch-activated Lethocerus muscle (Armitage et al. 1975) (again a pioneering study which is all the more remarkable since the binning of each contractile cycle into eight segments and its aggregation over hours of contraction needed to get a signal was controlled by a suitcase sized-PDP10 computer with just 8K of RAM and no hard disk!). On a sabbatical in San Francisco he published the seminal theoretical paper on fluorescence polarisation in muscle (Tregear and Mendelson 1975).

Richard was also a passionate educator. His animated film “What Makes Muscle Pull?” was made as long ago as 1971 (Tregear 1971) yet it remains a classic—and still correct—exposition of crossbridge cycling to this day. Although he rarely spoke at scientific meetings and he had only a few PhD students, he was a great promoter of scientific discussions in less formal situations. He was involved in reviving the Muscle Club Dinners in the 1970s and promoting the Alternative Muscle Club for student and postdocs, founded by his student, Maxine Clarke in 1980. He much preferred the small meetings and especially the Alpbach Workshops where his incisive questioning would catch unsuspecting postdocs off-guard and puncture the overambitious theorising of their supervisors. They could end up being signed up as collaborators by the end of the meeting.

His enthusiasm for scientific discourse culminated in the founding of the Journal of Muscle Research and Cell Motility with Chris Ashley in 1980. The first issue editorial starts with a passage that is quintessential Richard Tregear “This JournaI is intended for everyone interested in the problems of biological motion, whether in proteins or in plants. We believe that there are enough interests in common throughout this range of work to justify pulling all of them together in one place. The contents of the first issue confirm our prejudice—the range is enormous, the enthusiasm common to all. “

When John Pringle retired in 1985, the Oxford ARC unit closed and Richard moved to the ARC Institute at Babraham. Here he had fewer opportunities for muscle research. He took early retirement and became a ‘gentleman scientist’, travelling to laboratories near and far (especially Cambridge and Duke) for experimentation and debate until he was well into his 70s (Tregear et al. 2004; Wu et al. 2010).

We shall remember his ideas, style and enthusiasm. The Journal of Muscle Research he created has prospered for 40 years now and is a fitting legacy.

**Figure:**
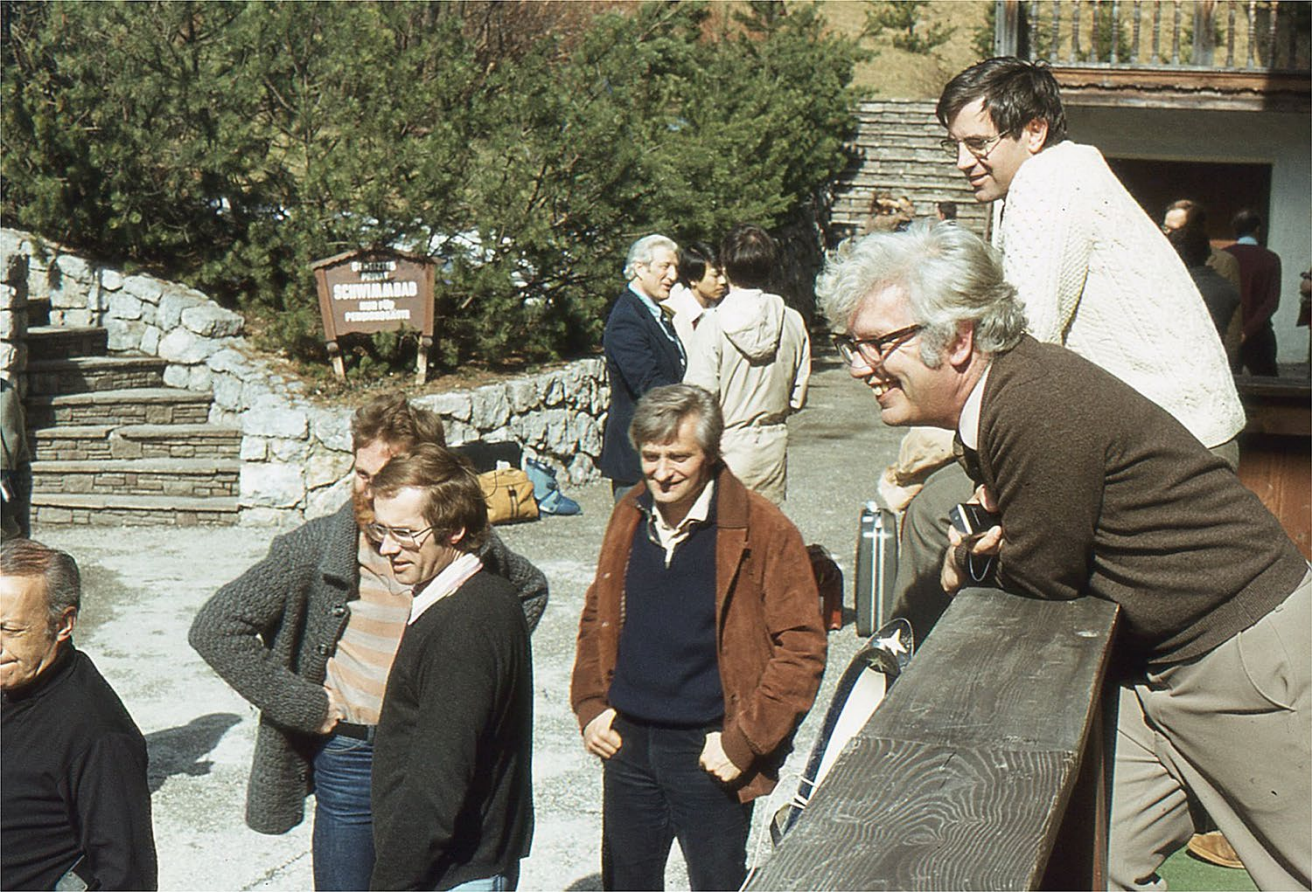
Richard Tregear at Alpbach, March 1974; photo by Clive Bagshaw
